# Effectiveness and prognostic factors of different minimally invasive surgeries for vertebral compression fractures

**DOI:** 10.1186/s12891-022-06125-8

**Published:** 2023-01-06

**Authors:** Wei Wang, Yi Liu, Hao Wan, LiangPing Zeng, ZongLi Peng, DanDan Yang, Kun Tian

**Affiliations:** 1 Department of Orthopaedics, The First Affiliated Hospital of Jiangxi Medical College, Shangrao, 334000 China; 2 Department of Orthopaedics, Fengcheng People’s Hospital of Jiangxi Province, Fengcheng, 331100 China; 3 Department of Orthopedics, Zhejiang Hospital of Traditional Chinese Medicine, Hangzhou, 310053 China

**Keywords:** Percutaneous vertebroplasty, Percutaneous kyphoplasty, Osteoporotic vertebral compression fracture, Curative effect, Factors affecting

## Abstract

**Background:**

The aging of China's population has led to an increase in the incidence rate of osteoporosis, which indirectly increases the risk of OVCF in osteoporosis patients. Low back pain is the main symptom of OVCF, and severe patients can further develop kyphosis. Although the conservative treatment of OVCF can effectively control the patient's condition, long-term bed rest will increase the risk of OVCF complications. Minimally invasive surgery is a common solution for OVCF.

**Methods:**

100 OVCF patients admitted to our hospital from January 2021 to January 2022 are selected for analysis and randomly divided into PVP group and PKP group, 50 cases in each group. The PVP group and the PKP group undergo PVP and PKP operations respectively. The differences in efficacy indicators and adverse reactions are compared, and the multivariate Logistic regression method is used to analyze the influencing factors of postoperative secondary fractures in patients with vertebral compression fractures.

**Results:**

Compared with the PVP group, the total effective rate of PKP group is significantly increased, and the VAS, ODI score, kyphotic Cobb Angle, lateral distribution rate of bone cement and bone cement leakage rate are significantly decreased (*P* < 0.05). Age ≥ 80 years old, female, glucocorticoid use, lateral distribution of bone cement and bone cement leakage are significantly higher in the proportion of secondary fractures and are independent risk factors for postoperative secondary fractures in patients with OVCF.

**Conclusion:**

PKP surgery has a higher efficacy in the treatment of OVCF patients, which can reduce the incidence of pain, adverse reactions and promote the recovery of kyphotic Cobb Angle. PKP surgery has a higher value in the treatment of OVCF. In addition, the influencing factors of secondary fracture after minimally invasive surgery in OVCF patients include age, gender, glucocorticoid use, bone cement distribution pattern, bone cement leakage, etc.

## Background

The increasing aging of the population in China leads to an increased incidence of osteoporosis, which indirectly increases the risk of OVCF in patients with osteoporosis. Low back pain is the main symptom of OVCF, and kyphosis is further developed in severe patients [1, 2].

Although the conservative treatment of OVCF can effectively control the patient's condition, long-term bed rest will increase the risk of OVCF complications. Common solutions of minimally invasive surgery for OVCF, including PVP and PKP, can achieve significant application effects, and PKP has a high acceptance in clinical OVCF [3, 4]. At this stage, there is little literature about the effect of PVP and PKP minimally invasive surgery on OVCF and the factors of secondary fracture in patients after surgery. Therefore, based on the comparative analysis of the effect of PVP and PKP minimally invasive surgery on OVCF patients, this study further discusses the factors that affect the secondary fracture after surgery, so as to provide a basis for the optimization of subsequent surgical programs and the improvement of prognosis.

The rest of this paper is organized as follows: Sect. 2 describes the work of this paper, and Sect. 3 analyzes the experimental results. Section 4 discusses the work of predecessors and this paper. Section 5 summarizes this paper.

## Methods

### General Information

A total of 100 patients with OVCF who are treated in our hospital from January 2021 to January 2022 are selected for study, and they are randomly divided into PVP group and PKP group, with 50 cases in each group. In the PKP group, the age ranges from 72 to 88 (82.81 ± 2.12) years, with 29 males and 21 females, 23 cases of technical secondary school and below, and 27 cases of college and above. The age of the PVP group is 72–88 (81.28 ± 2.54) years old, with 27 males and 23 females, 23 cases of technical secondary school and below, and 27 cases of college and above. There is no statistical difference in the data between the two groups (*P* > 0.05). All the included subjects meet the WHO diagnostic criteria for osteoporosis [5], and their imaging examination shows that the posterior vertebral wall is intact and they can tolerate PVP and PKP surgery, and all the patients and their relatives sign informed consent. Patients with pathological fracture of other reasons, fracture time more than 3 weeks or severe condition are excluded from the study.

## Experimental methods

### Clinical data collection

The self-designed clinical data sheet is used to collect the information of the study subjects, including age, gender, education level, bone cement distribution pattern, bone cement leakage rate, and glucocorticoid use.

### Surgical methods

PVP is performed in the PVP group. The specific measures are as follows: patients are placed in prone position after local anesthesia, the puncture area is disinfected with diluted betadine for three times, fluoroscopy is performed with C-arm X-ray machine to determine the puncture location, and conventional towel laying is performed. Under X-ray fluoroscopy, the puncture needle and collapsed endplate are placed in a parallel position, and the needle is slowly inserted at an oblique Angle to the sagittal plane. About 1/3 of the anterior vertebral body is reached through the cortical area of the posterior edge of the vertebral body, and the expansion tube is inserted to establish a working channel for bone cement perfusion. Pressure syringes are used to push PMMA bone cement mixed to dhone-like viscous shape into the affected vertebra slowly, and the injection of bone cement in the vertebra is observed with the assistance of X-ray machine fluoroscopy. The injection action is stopped before the bone cement is pushed to the posterior edge of the vertebra. Rotate the work sleeve until the bone cement solidifies and pull out the work sleeve. After disinfection, the patient is sutured. The patient is kept in a fixed position for 15 min, and returned to the ward when there is no abnormality.

PKP group is treated with PKP, the specific measures are as follows: Surgery preparation and prophase work and PVP set consistent, needle advance after placing balloon catheter and installation, to a balloon to inject the contrast agent and at the same time, see the perspective of vertebral height restoration after pressure stop and take out developer, perfusion bone cement and disinfection, suture operation in PVP set consistent, confirmed after back to the ward.

### Evaluation criteria

The evaluation criteria are as follows: (1) Efficacy evaluation criteria [6]: complete disappearance of clinical symptoms is regarded as cure. The clinical symptoms are obviously improved and the effect is remarkable. The improvement of clinical symptoms is effective. The clinical symptoms do not improve, even aggravated to no effect. The total effective rate is the cure rate, obvious efficiency and the proportion of effective rate in the total cases. (2) All patients are evaluated for pain by VAS, and a 10 cm long line segment is prepared. The two ends are marked with "no pain" and "extremely painful", and the patients are allowed to mark it online. Find the point that best represents its pain intensity, measure the distance to the "no pain" point and record its corresponding score. The total score is 10 points, and as the score increases, the patient's pain symptoms increase accordingly. (3) The ODI is used to evaluate the functional status from 9 dimensions: pain level, sexual life, self-care ability of daily living, lifting, walking, sitting, standing, sleep, social activities, and travel. The dimensions are scored according to 0–5 points, and the total score is 0–45 points. The functional impairment increases with the increase of the score. The three time points before surgery, 1 month after surgery, and 3 months after surgery are marked as T1 ~ T3.

### Statistical methods

Data are input into SPSS 26.0 software for statistical processing, measurement data are expressed as mean ± standard deviation(*x* ± *s*), *t*-test. Count data are represented by (%), *x*^*2*^ test. *F*-test is used for multiple groups of data. Repeated measurement analysis and spherical test (Mauchly) are used to analyze the data within the group. Multivariate logistic regression is used to analyze the influencing factors of postoperative secondary fractures in patients with vertebral compression fracture (*P* < 0.05), and the difference is statistically significant.

## Results

### Comparison difference in efficacy

Table 1 is the comparison of the differences in clinical efficacy. It is clearly evident from Table 1 that the PKP group has a higher overall response rate than the PVP group, and the data are statistically different (*P* < 0.05).

**Table 1:** Comparison of the differences in clinical efficacy(*n*, %)

Group	Cure	Excellence	Effective	Invalid	Total effective rate
PKP group	10 (22.00)	27 (50.00)	10 (22.00)	3 (6.00)	47(94.00)
PVP group	7 (16.00)	21 (38.00)	9 (34.88)	13 (26.00)	37(74.00)
*x*^*2*^					7.440
*P*					0.006

### Contrast of the VAS and ODI differences at different time points

Table 2 is the differences in VAS and ODI scores at different time observation points. It is clearly evident from Table 2 that all patients increase over time and their VAS and ODI scores remain decreased. and the PKP group had lower VAS and ODI scores, and the data are statistically different (*P* < 0.05).

**Table 2:** Differences in VAS and ODI scores at different time observation points(*x* ± *s*)

Group	Time point	VAS (score)	ODI(score)
PKP group	T1	7.38 ± 1.20	38.39 ± 4.22
	T2	4.30 ± 1.02	19.51 ± 3.08
	T3	2.57 ± 0.51	12.68 ± 1.62
PVP group			
	T1	7.35 ± 1.17	38.36 ± 4.19
	T2	5.39 ± 1.11	26.40 ± 3.13
	T3	3.68 ± 0.75	18.69 ± 1.86
*F* viewing time		432.414	441.334
*P* viewing time		< 0.001	< 0.001
*F* viewing time*block sort		513.308	587.232
*P* viewing Time*block sort		< 0.001	< 0.001

**Fig. 1:**
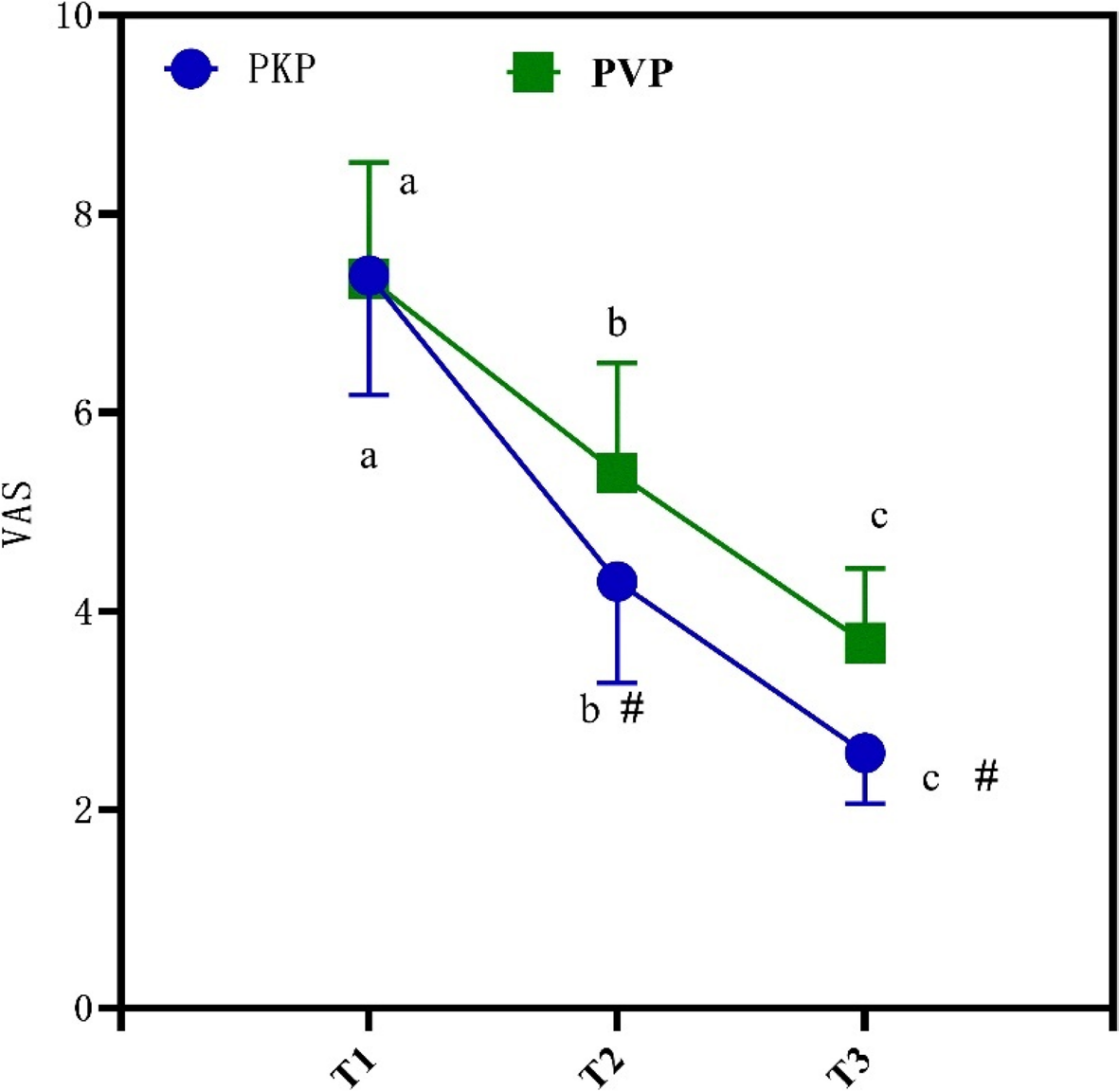
VAS, ODI score changes at different observation points at different times

Figure 1 is the VAS, ODI score changes at different observation points at different times. It is clearly evident from Fig. 1 that the PKP group has lower VAS and ODI scores, and the data are statistically different (P < 0.05).

### Comparing convex Cobb angles at different time points

Table 3 is the differences in kyphosis Cobb angle changes between observation points. It is clearly evident from Table 3 that all patients increase over time, the Cobb angle decrease and the PKP group has lower Cobb angle, and the data are statistically different (*P* < 0.05).

**Table 3:** Differences in kyphosis Cobb angle changes between observation points at different times(*x* ± *s*)

Group	Time point	Bulump Cobb angle(°)
PKPgroup	T1	23.38 ± 4.53
	T2	15.41 ± 3.12
	T3	8.48 ± 2.02
PVPgroup		
	T1	23.35 ± 4.49
	T2	19.10 ± 3.21
	T3	13.39 ± 2.21
*F* viewing time		432.444
*P* viewing time		< 0.001
*F* viewing time*block sort		522.338
*P* viewing time*block sort		< 0.001

### Comparing the distribution pattern of bone cement and bone cement leakage ratio

Table 4 is the comparison in cement distribution pattern and cement leakage rate. It is clearly evident from Table 4 that the PKP group has a lower lateral distribution rate of bone cement and bone cement leakage rate, and the data are statistically different (*P* < 0.05).

**Table 4:** Comparison in cement distribution pattern and cement leakage rate(*n*, %)

Group PKP group	Cement distribution pattern		Bone cement leakage
	Meso-position	LP	
PVP group	39(78.00)	11(22.00)	4 (8.00)
Group	23(46.00)	27(54.00)	12 (24.00)
*x*^*2*^	10.866		4.762
*P*	0.001		0.029

### Univariate analysis of the difference in secondary fracture status after minimally invasive surgery in patients with OVCF

Table 5 is the univariate analysis of the differences in secondary fractures after minimally invasive surgery in patients with OVCF. It is clearly evident from Table 5 that the proportion of secondary fractures at the age of 80 years, female gender, glucocorticoid use, lateral cement distribution pattern, and cement leakage is significantly higher (*P* < 0.05).

**Table 5:** Univariate analysis of the differences in secondary fractures after minimally invasive surgery in patients with OVCF(*n*, %)

Factor	*n*	Secondary fracture(*n* = 20)	No fracture(*n* = 80)	× *2*	*P*
Age (year)				6.510	0.011
< 80	40	3	37		
≥ 80	60	17	43		
Sex				4.474	0.034
Man	56	7	49		
Woman	44	13	31		
Degree of education				1.010	0.315
Technical secondary school and below	45	11	34		
College degree or above	55	9	46		
Glucocorticoid use				6.510	0.011
Yes	40	13	27		
Deny	60	7	53		
Cement distribution pattern				5.136	0.023
Lateral position	38	12	26		
Meso-position	62	8	54		
Bone cement leakage				15.644	< 0.001
Yes	16	9	7		
Deny	84	11	73		

### Multivariate logistic analysis of the factors influencing postoperative secondary fractures in patients with OVCF

Table 6 is the variable assignment table. Table 7 is the multivariate logistic analysis of the factors affecting postoperative secondary fractures in patients with OVCF. It is clearly evident from Tables 6 and 7 that the *P* < 0.05 factor is used as independent variable, and secondary fracture is performed as dependent variable: age 80 years, female gender, glucocorticoid use, cement lateral distribution pattern, and cement leakage are all independent risk factors for secondary fracture in patients with OVCF.

**Table 6:** Variable assignment table

Factor	Variable name	Assignment
Age (year)	*X1*	≥ 80 = 1, < 80 = 2
Sex	*X2*	Female = 1, male = 2
Glucocorticoid use	*X3*	Yes = 1, No = 2
Side-position distribution pattern of bone cement	*X4*	Side position position = 1, median position = 2
Bone cement leakage	*X5*	Yes = 1, No = 2
Postoperative secondary fracture	*Y*	Yes = 1, No = 2

**Table 7:** Multivariate logistic analysis of the factors affecting postoperative secondary fractures in patients with OVCF

Bone cement leakage	*B*	*S.E*	*Wald*	*P*	*OR*	*95%CI*
Distribution of cement (lateral)	1.100	1.265	4.611	0.024	0.241	0.045 ~ 0.898
Glucocorticoid use	3.430	1.283	7.035	0.007	0.133	0.019 ~ 0.648
Gender (female)	1.242	0.415	9.063	0.001	0.087	0.018 ~ 0.423
Age (80 years old)	3.328	1.172	7.024	0.005	0.132	0.010 ~ 0.644
Influencing factor	1.098	1.274	4.619	0.033	0.230	0.052 ~ 0.887

**Fig. 2:**
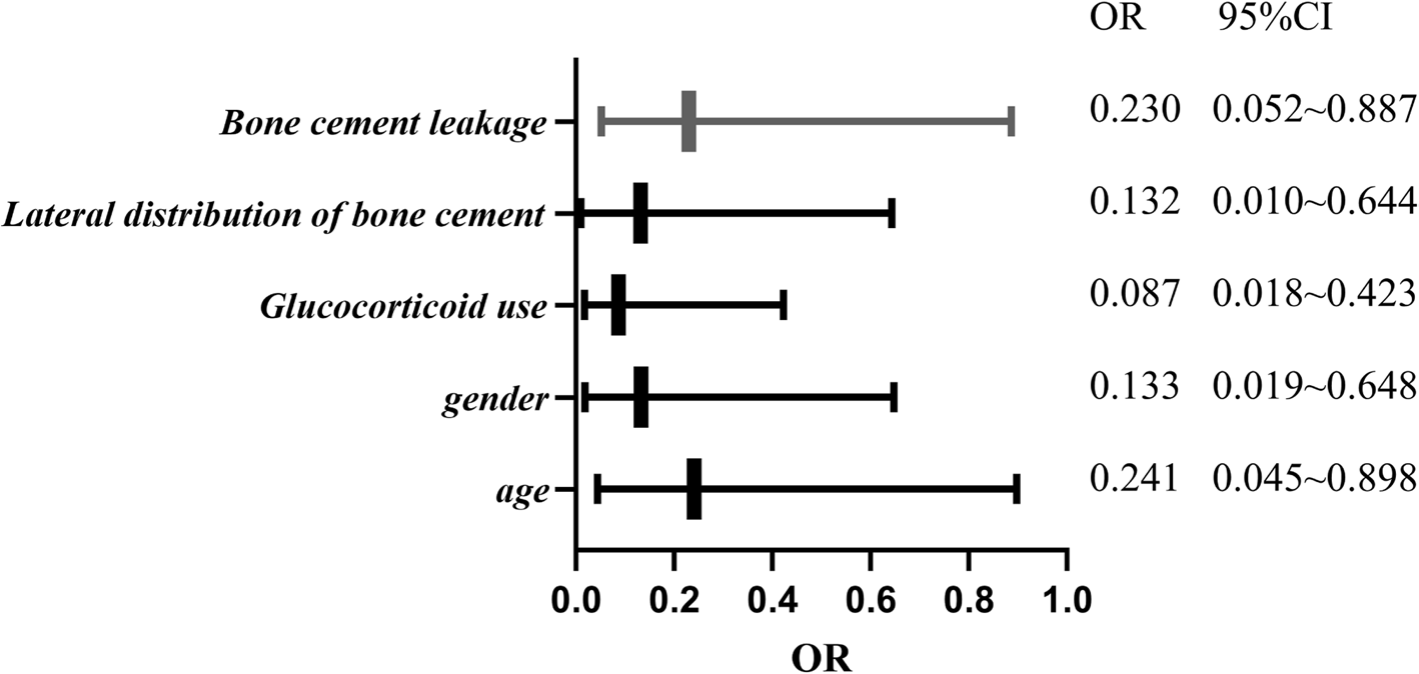
Forest map of influencing factors of postoperative secondary fractures in patients with OVCF

Figure 2 is the forest map of influencing factors of postoperative secondary fractures in patients with OVCF.

## Discussion

OVCF mainly occurred in the anterior and middle column of the vertebral body of the spine. The anterior 2/3 vertebral body, anterior 1/2 annulus and anterior longitudinal ligament were the main components of the anterior column, while the posterior 1/3 vertebral body, posterior longitudinal ligament and posterior 1/2 annulus were the main components of the middle column. The compression degree of the anterior and middle columns was more serious than that of the posterior column. The sagittal multi X-ray imaging results of OVCF patients had the characteristics of "being wedged back and forth". The abnormal increase of Cobb angle was a typical manifestation of OVCF patients. At the same time, the kyphosis would aggravate the patients. If the patients failed to go to the hospital in time and compress the vertebral body height, it would further aggravate the abnormal spinal mechanical conduction and persistent back pain. Some patients would have serious complications, such as nerve root injury. It seriously threatened the life safety and health of patients [7]. The application of PKP surgery in the treatment of OVCF is helpful to restore vertebral height and reduce body pain, and PKP surgery has been validated in a number of studies on OVCF-related diseases [8].

In a previous study, it was pointed out that PVP surgery could be polymethyl methacrylate bone cement perfusion vertebral body fracture directly, and effectively reduce OVCF patients with low back pain symptoms, but the operation was affected by the perfusion pressure, the deficiency of the bone cement leakage happens after infusion [9]. PKP held through balloon expansion can promote the vertebral height restoration and vertebral body fracture reduction. At the same time, it could improve the postoperative Cobb Angle and the height of anterior vertebral body. The results of this study were basically consistent with the conclusions of previous studies. In this way, the height of the vertebral body could be restored [10]. The results of this study showed that the distribution morphology of bone cement and the leakage rate of bone cement in the PKP group were better than those in the PVP group, which were consistent with the results of previous studies [11]. The reason might be that PKP could narrow the fracture gap and compact the surrounding bone by compressing it, thereby effectively reducing the risk of leakage of bone cement injected into the internal bone, and making the bone cement evenly distributed.

The results of this study showed that age ≥ 80 years old, female, glucocorticoid use, lateral distribution pattern of bone cement, and bone cement leakage were independent risk factors for postoperative secondary fracture in patients with OVCF, which were basically consistent with the results of previous studies [12, 13]. The mechanism analysis was as follows: The new vertebral fracture after PVP was related to the patient's age. With the growth of age, the bone loss of patients increases and estrogen abnormally decreased leading to the weakening of osteoblast function, thus reducing bone strength and increasing fracture risk [14]. The risk of postoperative new vertebral fractures in female patients was higher than that in male patients, which might be due to the fact that most patients with spinal compression fractures are elderly, and the estrogen level in female patients at this stage was significantly reduced, which would further affect bone metabolism and increase bone loss, resulting in bone hardness reduction and fracture [15].

## Conclusion

In conclusion, PKP surgery in the treatment of OVCF patients can improve the efficacy, reduce the incidence of pain and adverse reactions, and improve the kyphotic Cobb Angle of patients. PKP surgery has promotion value in the treatment of OVCF. Many factors, such as age, gender, glucocorticoid use, bone cement distribution and bone cement leakage, can increase the risk of secondary fracture after minimally invasive surgery in OVCF patients. In this study, the reduction of VAS, ODI, and Cobb Angle in the PKP group is greater than that in the PVP group, which further suggests that PKP treatment can improve the efficacy of minimally invasive surgery in patients with OVCF, and reduce the symptoms of patients with low back pain, limb dysfunction, and abnormal increase of Cobb Angle. The main mechanism of its effect is speculated as follows: PKP holding operation mainly destroys the vertebral body and peripheral nerve endings through skin perfusion and pedicle bone cement. The analgesic effect is successively in the sleeve when the vertebral body is compressed, the balloon expands, can rise and reposition, support and compress the vertebral body and other multiple effects. At the same time, the bone cement can also support and strengthen the vertebral body, and promote the rapid convergence of vertebral body small fractures.

## Data Availability

The datasets used and/or analyzed during the current study are available from the corresponding author on reasonable request.

## Abbreviations

OVCF: Osteoporotic Vertebral Compression Fracture
PVP: Percutaneous Vertebral Plasty
PKP: Percutaneous Kyphoplasty
PMMA: Polymethyl Methacrylate
VAS: Visual Analogue Scale
ODI: Oswestry disability index
